# SSR marker development and intraspecific genetic divergence exploration of *Chrysanthemum indicum* based on transcriptome analysis

**DOI:** 10.1186/s12864-018-4702-1

**Published:** 2018-04-25

**Authors:** Zhengzhou Han, Xinye Ma, Min Wei, Tong Zhao, Ruoting Zhan, Weiwen Chen

**Affiliations:** 1 Research Center of Chinese Herbal Resource Science and Engineering, Guangzhou University of Chinese Medicine; Key Laboratory of Chinese Medicinal Resource from Lingnan (Guangzhou University of Chinese Medicine), Ministry of Education; Joint Laboratory of National Engineering Research Center for the Pharmaceutics of Traditional Chinese Medicines, Guangzhou, 510006 People’s Republic of China; 2 China Resources Sanjiu Medical & Pharmaceutical Co., Ltd, Shenzhen, 518110 Guangdong China

**Keywords:** *Chrysanthemum indicum*, Germplasm identification, Intraspecific genetic divergence, SSR marker, Transcriptome

## Abstract

**Background:**

*Chrysanthemum indicum* L., an important ancestral species of the flowering plant chrysanthemum, can be used as medicine and for functional food development. Due to the lack of hereditary information for this species and the difficulty of germplasm identification, we herein provide new genetic insight from the perspective of intraspecific transcriptome comparison and present single sequence repeat (SSR) molecular marker recognition technology.

**Results:**

Through the study of a diploid germplasm (DIWNT) and a tetraploid germplasm (DIWT), the following outcome were obtained. (1) A significant difference in Gene Ontology (GO) and Kyoto Encyclopedia of Genes and Genomes (KEGG) annotations for specific homologous genes was observed using the OrthoMCL method for the identification of homologous gene families between the two cytotypes. Ka/Ks analysis of common, single-copy homologous family members also revealed a greater difference among genes that experienced positive selection than among those experiencing positive selection. (2) Of more practical value, 2575 SSR markers were predicted and partly verified. We used TaxonGap as a visual tool to inspect genotype uniqueness and screen for high-performance molecular loci; we recommend four primers of 65 randomly selected primers with a combined identification success rate of 88.6% as priorities for further development of DNA fingerprinting of *C. indicum* germplasm.

**Conclusions:**

The SSR technology based on next-generation sequencing was proved to be successful in the identification of *C. indicum* germplasms. And the information on the intraspecfic genetic divergence generated by transcriptome comparison deepened the understanding of this complex species’ nature.

**Electronic supplementary material:**

The online version of this article (10.1186/s12864-018-4702-1) contains supplementary material, which is available to authorized users.

## Background

*Chrysanthemum indicum* L., a perennial herbaceous species of the Asteraceae family, originates from and is currently distributed mainly in East Asia [1]. In China, the dried inflorescence of *C. indicum* has been used as medicine for over 2000 years. The taste, meridian tropism, and efficacy of this medicine are documented in the 2010 edition of Chinese Pharmacopoeia [2] as follows: bitter, acrid, and slightly cold; liver and heart; and clearing heat, detoxifying, purging fire and calming the liver, respectively. With the recent advances in phytochemical and pharmacological research, *C. indicum* is being increasingly used clinically, which has impacted the once rich wild resources. In 2012, the Ministry of Health of the People’s Republic of China confirmed that *C. indicum* was on the list of items available for functional food, which increased its potential market demand. However, *C. indicum* is still in a “complex” state [1, 3, 4]; it has diverse morphological features without distinct boundaries, and genetic variation exists both within and among populations [1, 5–9], making germplasm identification difficult. This obstacle must be overcome to identify wild medicinal herbs and determine the authenticity and purity of cultivated varieties. The use of molecular markers can prevent the interference of confusing phenotypes and directly identify specific genotypes at the genetic level [10, 11], and such markers should be developed as an effective new approach for identifying *C. indicum* germplasms.

*C. indicum* has also received attention for another reason: numerous studies have shown that it is an important ancestral species of the plant that produces the well-known flower chrysanthemum [12]. Thus, *C. indicum* has major theoretical and practical significance for addressing various problems of chrysanthemum, including origin and evolution determination, identification and classification, and variety breeding. However, the genetic characteristics, especially the intraspecific divergence, of this species complex remain poorly understood [3, 4, 13]. It has already been shown that due to frequent natural hybridization, even interspecific differences between *C. indicum* and several congeneric species are indistinguishable by genomic in situ hybridization (GISH) [14]. In addition, a previous study reported that based on both low overall genetic variation and high individual phenotypic diversity, *C. indicum* has experienced rapid adaptive radiation [6]. These reports suggest the potential challenge in characterizing the intraspecific genetic divergence of this species. It is possible that a more detailed identification of homologous genes within and between different operational taxonomic units (OTUs, which may refer to such items as germplasm, population, and species) based on a sequence similarity algorithm may help to reveal its genetic divergence. In addition, comparison of the types and intensities of natural selection for common homologous genes shared by different OTUs might lead to a better understanding of the inherited variation from an evolutionary point of view.

The primary aim of the present study was to carry out the molecular identification of *C. indicum* germplasms to develop and validate simple sequence repeat (SSR) primers and to subsequently analyze their discriminative power. The secondary aim of this study was to gain new insight into the genetic characteristics of *C. indicum* by comparing specific and common homologous genes between germplasms of different cytotypes.

## Methods

### Materials

**Fig. 1:**
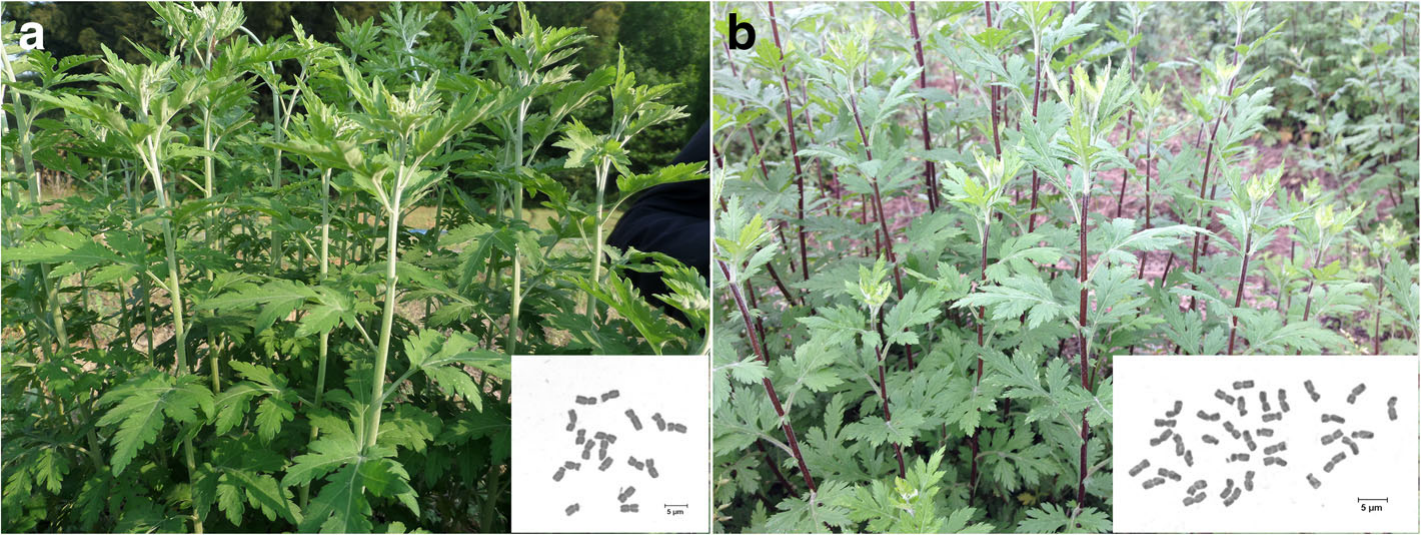
*C. indicum* plants of two cytotypes. **a** diploid, 2n = 2× = 18, marked as DIWNT; (**b**) tetraploid, 2n = 4× = 36, marked as DIWT. (bar = 5 μm)

Plant samples for RNA-seq were preserved by asexual cutting propagation at the *C. indicum* germplasm resource nursery of China Resources Sanjiu Medical & Pharmaceutical Co., Ltd., Yangxin County, Hubei Province, China. Fresh samples of healthy tissues were taken from different parts (roots, stems, leaves, buds, initial blooms, and full blooms) of two *C. indicum* germplasms and immediately frozen in liquid nitrogen. The samples were mixed with equivalent qualified RNA for transcriptome sequencing. Diploid germplasm was labeled DIWNT; tetraploid germplasm was labeled DIWT (Fig. 1). Partial information about morphological characteristics of these 2 representative *C. indicum* germplasm could be found in the Additional file 1.

A total of 86 populations (separated from each other by at least 100 m) of *C. indicum* were sampled across the wild and planting bases in 5 provinces of China (Additional file 2). Among then, 23 from Hubei province; 16 from Henan province; 20 from Anhui province; 15 from Guangdong province; 12 from Guangxi province. Ten individuals were collected but only one selected from each population for molecular marker test. Young leaves of each individuals were immediately dried with silica gel before transportation and storage. All voucher specimens were deposited at the herbarium of China Resources Sanjiu Medical & Pharmaceutical Co., Ltd.

### Methods

#### Transcriptome sequencing, de novo assembly and function Annonation

Total RNA samples of acceptable purity and concentration were obtained, and library construction was then performed. mRNA was enriched using oligo dT beads, and the purified mRNA was fragmented. First-strand cDNA was synthesized by using reverse transcriptase, and double-stranded cDNA was synthesized using the first-strand cDNA as a template. The ends of the double-stranded cDNA were repaired, and a “polyA” tail was added to the 3′ end. Both ends of the fragments were connected to adaptor sequence and the cDNA fragments were purified from gels. The library was amplified using high-fidelity polymerase, and the quality of library construction was checked. Finally, sequencing was performed using the Illumina HiSeq 2000 High-Throughput Sequencing platform (Illumina, Inc., USA).

Raw sequence data were subjected to yield statistics, and clean reads (Accession: SRX2493247 and SRX2493239) were obtained after filtering adaptor and reads with ambiguous ‘N’ bases and base quality less than Q30. Trinity [15] was used to assembly the clean reads into contigs and contigs were clustered and further assemblied into transcripts. Functional annotation was conducted by aligning (BLASTx, E-value ≤1 × 10^− 5^) the unigenes to public protein databases including National Center for Biotechnology Information (NCBI) nr, SwissProt, Kyoto Encyclopedia of Genes and Genomes (KEGG), and Clusters of Orthologous Groups (COG). Blast2GO [16] and WEGO [17] were used for Gene Ontology (GO) analysis with default settings.

#### Homologous gene identification

We mixed the unigene data obtained from the transcriptomes of the two *C. indicum* germplasms to form a pan-transcriptome. All-against-all pairwise alignments of protein sequences were performed by blastp (E-value≤1 × 10^− 7^). The alignment results were clustered into OrthoMCL clusters, i.e., into gene families, using the OrthoMCL method [18]. Each cluster contained more than two homologous genes. Homologous genes specific and common to the germplasms were identified based on their number and origin in each family.

#### Evolutionary pressure analysis

To determine the presence of selection pressure on protein-coding genes, we calculated the ratio between non-synonymous (Ka) and synonymous (Ks) substitution rates, i.e., Ka/Ks, of single-copy gene family members common to the two germplasms using KaKs_Calculator (https://sourceforge.net/projects/kakscalculator2/). Genes for which Ka/Ks was not calculated and those with Ka/Ks > 0.1 were excluded from subsequent analyses. Based on the calculation results, we classified the genes as follows: Ka/Ks > 1 indicates genes under strong positive selection (that had previously experienced positive selection); 1 > Ka/Ks > 0.5 indicates genes under weak positive selection (that are currently experiencing positive selection). GO annotation analysis was conducted on genes that had previously experienced or are currently experiencing positive selection in different germplasms.

#### SSR primer prediction and PCR experiments

All unigenes generated by deep transcriptome sequencing of diploid germplasm (DIWNT) were screened for SSRs using a Perl script known as MIcroSAtellite (MISA, http://pgrc.ipk-gatersleben.de/misa). The following screening criteria were used: length of SSR repeat motifs of 2–6 bp; frequency of dinucleotide repeats ≥6; frequency of trinucleotide repeats ≥5; frequency of tetra-, penta-, and hexanucleotide repeats ≥4; and distance between two SSR sequences ≥100 bp. Primer pairs were designed using Primer3 (http://primer3.sourceforge.net/) using the following criteria: predicted product size of 100–275 bp, 40–60% GC, optimum primer length of 22 bp, and melting temperature of 55–60 °C.

The molecular markers were validated as follows. (1) Genomic DNA was extracted from dry leaf samples using the commercial kit Plant DNA Isolation Reagent D9194 (Takara Biotechnology (Dalian) Co., LTD, China). (2) Predicted primers were selected at random for polymerase chain reaction (PCR) amplification of partial samples, and primers that produced bands were identified and screened by agarose gel electrophoresis. (3) The initially screened forward primers were re-amplified after fluorescence labeling with GeneScan™-500 LIZ® (Applied Biosystems, USA). The amplification products were separated by capillary electrophoresis using an ABI Prism® 3730 Genetic Analyzer (Applied Biosystems, USA). Primers with good polymorphisms were further screened. (4) Fluorescent primers with good polymorphisms were used to amplify all samples. Raw data (.FSA) were exported using GeneMarker v2.2.0 (SoftGenetics LLC., USA).

Each 15-μl PCR reaction contained 1.5 μl 10 × ExTaq buffer, 1 μl 2.5 mM deoxynucleotides (dNTPs), 0.5 μl 10 mM forward primer, 0.5 μl 10 mM reverse primer, 1 μl DNA template, 0.25 μl EasyTaq, and 10.25 μl H_2_O. The following PCR conditions were used: 95 °C for 5 min; 10 cycles of 95 °C for 20 s, 55–60 °C for 20 s, and 72 °C for 30 s; 25 cycles of 95 °C for 20 s, 55 °C for 20 s, and 72 °C for 30 s; and a final step at 72 °C for 30 min. The temperature was then held at 12 °C.

#### SSR data processing and germplasm identification capacity analysis

Raw data (.FSA) were imported into GeneMarker for manual verification and validation of SSR amplification bands. We classified the amplification results into different projects based on the type of primers. A dendrogram view of all samples in a project was created using the clustering analysis module to show the polymorphisms in the amplification products from a particular primer; the distance measure and the linkage type used were “percentage of same genotypes” and “single”, respectively. A similarity matrix table was prepared using the “clustering report” module of GeneMarker for subsequent analysis of species identification capacity by TaxonGap [19]. TaxonGap compares the genetic distance between OTUs (herein referred to as germplasm) to directly show the identification efficiency of different primers, namely, SSR loci, in the range of the experimental samples. Those OTUs that can be separated from their nearest neighbor indicate that a unique “DNA band fingerprint” unlike any other germplasm can be amplified by a specific primer.

## Results

### Transcriptome data statistics, assembly, evaluation and functional annotation

We obtained high-quality transcriptome data from the germplasm of two cytotypes of *C. indicum*, diploid (DIWNT) and tetraploid (DIWT) (Table 1). The percentage of bases with a quality score no less than 20 for DIWNT and DIWT was 96.6% and 97.1%, respectively. Using the short-read assembler Trinity, we obtained 42,023 DIWNT unigenes, with a mean transcript length of 727.72 bp, and 46,049 DIWT transcripts, with a mean transcript length of 784.89 bp. The GC content of the transcriptome reads from *C. indicum* the diploid and tetraploid germplasms was 46.81% and 44.89%, respectively. Unigenes were compared to protein sequence databases including NCBI nr, SwissProt, KEGG and COG. The final 31,347 DIWNT transcripts and 33,915 DIWT transcripts were annotated as known function, accounting for approximately 3/4 of the total number of transcripts from each germplasm. Interestingly, with regard to the numbers of total reads, total unigenes, annotated genes in each database, and total annotated genes, the tetraploid germplasm exhibited values only approximately 10% (8.1–13.0%) greater than those of the diploid germplasm.

**Table 1:** Summary of transcriptome information for diploid and tetraploid *C. indicum* germplasms

Samples	Total Reads	GC percentage	Total Unigenes	Average length (bp)	Annotation genes					Annotation ratio
					Nr	SwissProt	COG	KEGG	Total	
Diploid	50,879,590	46.81%	42,023	727.72	31,214	23,181	11,144	9947	31,347	74.60%
Tetraploid	56,522,734	44.89%	46,049	784.89	33,742	25,197	12,591	10,992	33,915	73.60%

### Comparison of specific expressed homologous genes between the two germplasms

**Fig. 2:**
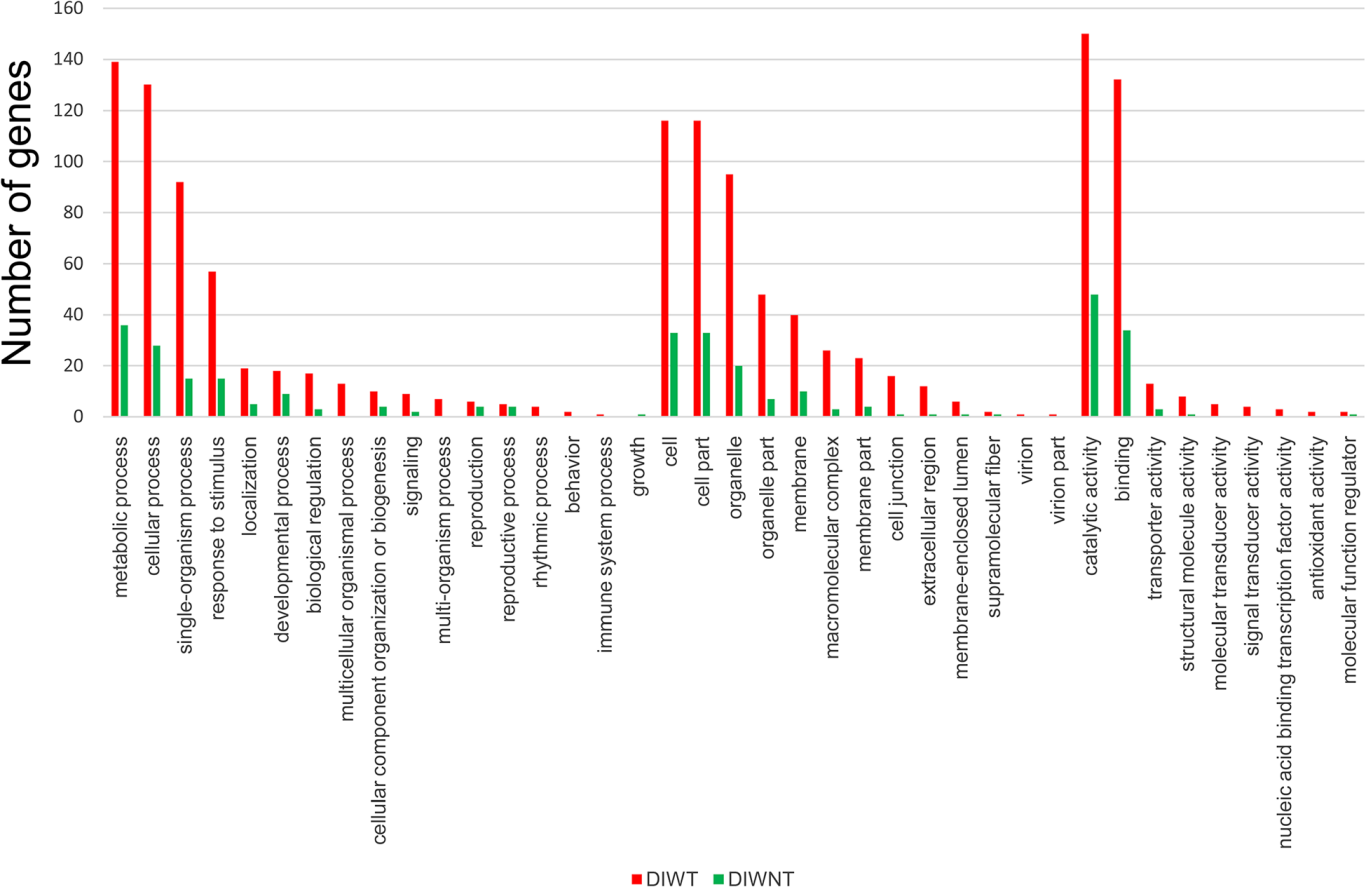
GO annotation comparison of specific homologous unigenes between diploid and tetraploid *C. indicum* germplasms

Among the 18,056 homologous gene families identified by the OrthoMCL method, 17,406 (96.40%) were common to both germplasms, whereas 208 (1.15%) and 442 (2.45%) families were specific to DIWNT and DIWT, respectively. We compared the GO functional annotations of 470 unigenes in 208 DIWNT-specific gene families and 1055 unigenes in 442 DIWT-specific gene families. As shown in Fig. 2, except for one gene of the diploid germplasm that was annotated with the “growth” GO term, the tetraploid germplasm exhibited a greater number of annotated genes in the remaining 38 GO terms; among these, 27 were shared by the two germplasms, and 11 were specific to the tetraploid germplasm. Some valuable molecular hints to the differential adaptability were also found. For an example, in the “response to stimulus” entry of the biological process, the number of enriched specific genes for the tetraploid germplasm was almost four times greater than that for the diploid germplasm (57/15).

**Fig. 3:**
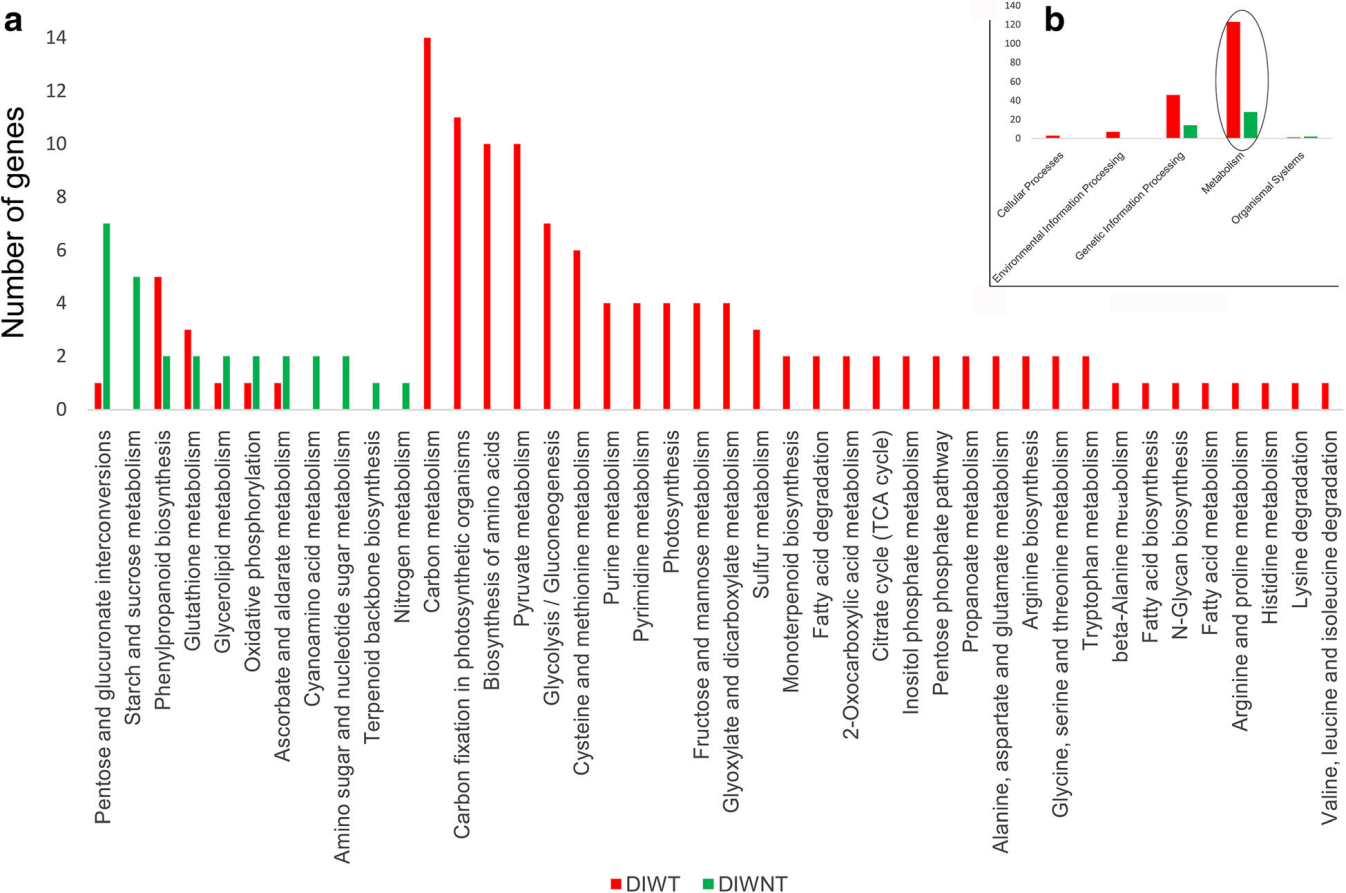
KEGG annotation comparison of specific homologous unigenes between diploid and tetraploid *C. indicum* germplasms. **a** KEGG-C-class annotation of genes annotated to the metabolism group; (**b**) KEGG-A-class annotation of all genes

Additionally, we compared the KEGG annotations of specific homologous genes between the different germplasms, further revealing differences in the complex biological behaviors of the various germplasms. The graph based on KEGG A class annotation (Fig. 3b) shows that the greatest number of unigenes (123 from DIWT and 28 from DIWNT) were annotated as the “metabolism” group, with the most impressive differences. The “metabolism” group was further subdivided into 42 pathways (Fig. 3a). Among these pathways, 6 were commonly shared by the two germplasms, 5 were diploid-specific pathways, and 31 were tetraploid-specific pathways. Of the 31 tetraploid-specific pathways, the well-marked 21 pathways included 3 energy metabolism pathways represented by photosynthetic carbon sequestration, 8 carbohydrate metabolism pathways represented by pyruvate generation and glycolysis, and 10 amino acid metabolism pathways represented by cysteine and methionine metabolism.

### Positive selective pressure on homologous genes common to the two germplasms

**Fig. 4:**
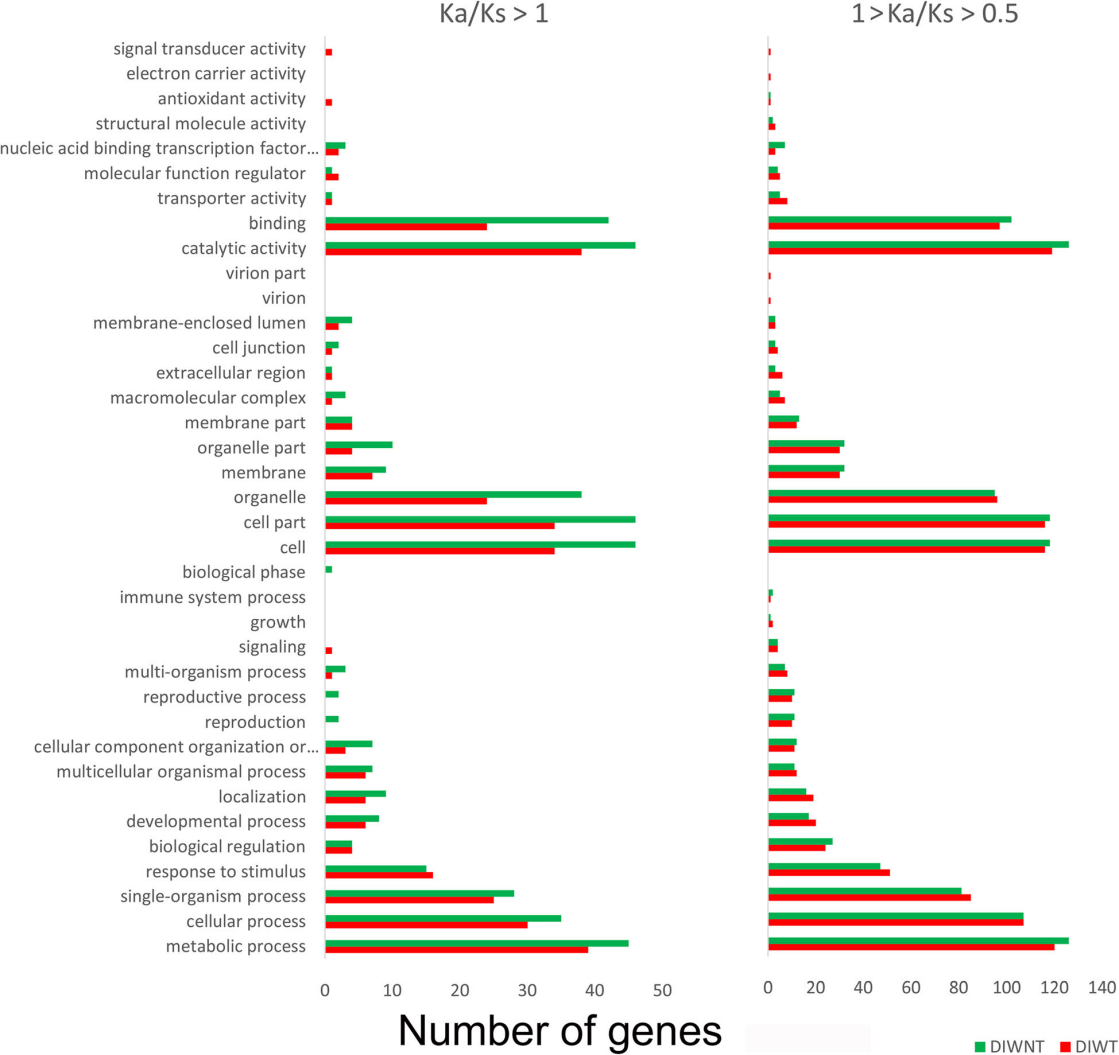
Comparison of GO annotations of positively selected genes between diploid and tetraploid *C. indicum* germplasms. Ka/Ks > 1, genes that have experienced positive selection; 1 > Ka/Ks > 0.5, genes that are experiencing positive selection

First we selected 15,646 single-copy homologous gene families common to the two germplasms (89.9% of the total homologous families common to the two germplasms) for Ka/Ks value calculation; and next we compared GO annotation information for genes that had previously experienced positive selection with that of genes currently experiencing positive selection. As shown in Fig. 4, Ka/Ks > 1 was calculated for 422 DIWNT genes, a value higher than the number of 318 calculated for DIWT genes. The diploid germplasm generally showed a large number of genes in the 25 GO terms shared by the two germplasms, such as “metabolic process”, “cell”, and “catalytic activity”. In contrast, 1 > Ka/Ks > 0.5 was determined for 1149 and 1144 genes of the diploid and tetraploid germplasms, respectively, and more similar GO annotation features were observed.

### Mining and validation of SSR molecular markers

In this study all 42,023 unigenes in the DIWNT transcriptome were scanned by MISA software (Table 2), and 2575 SSR markers present in 2302 genes were identified. The top three repeat types were trinucleotide SSRs (1452), dinucleotide SSRs (714) and tetranucleotide SSRs (271), which accounted for 94.6% of the total SSRs. Within the identified motif sequences, the top three most frequent SSRs were AC/GT (16.9%), ATC/ATG (14.3%), and ACC/GGT (14.1%).

**Table 2:** Summary of SSRs identified in the transcriptome of diploid *C. indicum* germplasms

Statistical Items	Numbers
Total number of sequences examined	42,023
Total size of examined sequences (bp)	30,580,946
Total number of identified SSRs	2575
Number of SSR-containing sequences	2302
Number of sequences containing more than 1 SSR	239
Number of SSRs present in compound formation	138
Di-nucleotide	714
Tri-nucleotide	1452
Tetra-nucleotide	271
Penta-nucleotide	58
Hexa-nucleotide	80

**Fig. 5:**
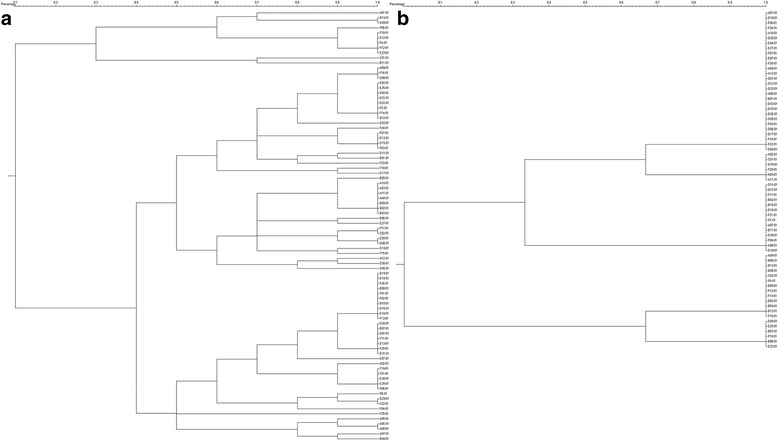
Cluster analysis of 86 *C. indicum* germplasm samples based on two SSR markers. **a** primer #1; (**b**) primer #4. The dendrogram shows the genetic similarity between 86 individuals. The scale on the top indicates the number of similar genotypes divided by the total number of genotypes. No PCR amplification signals or suspected signals were excluded

Sixty-five predicted primer pairs were selected at random and validated by two rounds of PCR amplification with partial samples, and 20 working primer pairs (Additional file 3) were chosen for the final experiment with all 86 samples. Based on GeneMarker software identification and manual confirmation, and despite the loss of some data, the overall result showed that according to different polymorphisms in the amplified bands, the effects of various primers on cluster analysis were markedly different for the same sample group, as typically and partially indicated in Fig. 5.

### Germplasm identification capacity of SSR molecular markers

**Fig. 6:**
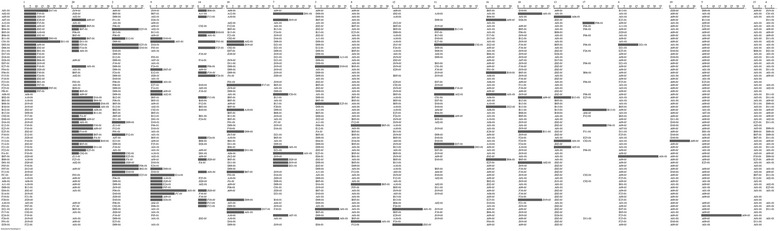
Schematic of identification capacity analysis of 20 SSR primers in 70 *C. indicum* germplasm samples. The left column shows a list of germplasm samples with unique genotypes. The matrix on the right presents the different separability (i.e., distance, presented as a dark gray horizontal bar) values of the same germplasms as rows and different biomarkers (SSR primers) as columns. Biomarkers are ranked by a combination of amplification and identification success rates. For each germplasm and each biomarker, the nearest neighbor is listed on the right side of the corresponding dark gray bar

According to the experimental results, 16 germplasms could not be distinguished by any of the 20 primer pairs, and their genotypes were thought to not be unique; these germplasms were omitted from subsequent analyses (the complete schematic is shown in Additional file 4). For the remaining 70 germplasms, the 20 primer pairs produced 1536 effective and credible PCR products, with the success rates of PCR amplification and identification differing among the primers. Overall, it was necessary to combine 10 primer pairs (primers #1, 20, 2, 9, 14, 10, 7, 3, 8, and 12) to distinguish all of the germplasm samples (Fig. 6). Among the 10 primers, combining four of them (primers #1, 20, 2 and 9) successfully identified 62 samples (88.6% of the total samples) and produced the best performance/cost ratio. Therefore, these four primers were preferred for the subsequent development of the DNA fingerprints. Moreover, it should be noted that in this study, two or more sets of specific bands were amplified by two or more sets of primers from each of 51 of 70 samples, facilitating cross-validation of the reliability of *C. indicum* germplasm-specific electronic identity card compilation.

## Discussion

So far there are still many unsettled problems about intraspecific differences in *C. indicum*, to which we had revealed some clues in this study through comparative transcriptome analysis of representative germplasm. (A) The GC contents of the transcriptional data from diploid and tetraploid germplasms were similar, close to that of *Chrysanthemum nankingense* (45.05%) [20] which was once considered as an infraspecies of *C. indicum*, but notably distinct from that of *Chrysanthemum morifolium* (37.32%) [21] indicating great differences between the ancestral species and the progeny species. (B) Regardless of the relationship between the two germplasm, there was good reason to believe the existence of the phenomena of polyploid genome shock and transcriptome shock [22, 23], which would make the differences in final expressed genes number not as dramatic as chromosome “doubling”. (C) Some molecular mechanisms were also suggested. For instance, more number of enriched specific genes in the “response to stimulus” entry of the biological process from tetraploid germplasm may explained the phenomenon that it is more widely distributed and more adaptable. When KEGG information analyzed, results intimated that the more specifically expressed basic metabolism pathways may be another crucial internal cause underlying the higher adaptability and broader distribution of tetraploids than diploids. (D) Microscopic-level changes in adaptive evolution indicate that genes are subjected to positive selection [24], which occurs with species (or germplasm) specificity. So the knowledge of the overall distribution characteristics of positively selected genes in different germplasms of *C. indicum* is important for understanding intraspecies variation. The result of Ka/Ks value calculation which provided a powerful tool for quantifying molecular evolution [25] indicated that, more attention to homologous single-copy genes having experienced positive selection should be payed for understanding intraspecific differences in *C. indicum* at the evolutionary scale.

An increasing number of successful examples have supported the strategy of using transcriptome data to predict SSR molecular markers [26], which has inspired improvement in the techniques available for *C. indicum* germplasm characterization. Research on the development of SSR markers in this work was merely a start. Follow-up study should include the direct selection of high-efficiency transferable SSR primers from the closely related species, such as *C. morifolium* [27, 28], as the other fast-forward strategy for *C. indicum* intraspecies characterization [29]. In addition, the application of SSR markers for diversity and DNA fingerprinting analyses has recently been reported in several plant species [20, 27, 28, 30–32], and the economy and practicality of this technique is determined by the ability to choose fewer but more effective primers. Unlike other jobs [27] that required artificial statistics, this study used TaxonGap software to form statistical data “automatically”, compare the identification efficiency of multiple molecular markers and screen samples with a “unique genotype”. Moreover, this software can visualize all the distances between/among its nearest neighbors in each sample under different candidate molecular loci in the same interface, thus simultaneously locating detailed data and evaluating the overall characterization effect.

## Conclusions

Summing up, we carried out a referential attempt toward the better understanding of the nature of *C. indicum* through transcriptome comparison between the two cytotypes. Specific and commom homologous genes identified by the OrthoMCL method could be used to find the special features of particular germplasm and the intraspecific difference in the perspective of evolutionary biology. Furthermore, we enriched germplasm identification method of this complex species by developing SSR marker technique, which was more objective than morphological way, and could provide stronger foundation for this traditional medicinal material to enter the new era of herbal genomics [33].

## Additional files

Additional file 1: Some information extracted from the doctoral thesis about phenotypic difference betwwen two *C. indicum* germplasms. (DOCX 2701 kb)

Additional file 2: Table Information for 86 *C. indicum* samples. (DOC 121 kb)

Additional file 3: Table Information of 20 selected primers. (XLSX 10 kb)

Additional file 4: Figure Schematic of identification capacity analysis of 20 SSR primers in all 86 *C. indicum* germplasm samples. (TIF 1428 kb)

## Abbreviations

COG: Clusters of Orthologous Groups
GO: Gene Ontology
KEGG: Kyoto Encyclopedia of Genes and Genomes
NCBI: National Center for Biotechnology Information
nr: Non-redundant protein sequences
OTU: Operational Taxonomic Units
PCR: Polymerase Chain Reaction
SSR: Single Sequence Repeat
