# Supplementary material for: SSR marker development and intraspecific genetic divergence exploration of Chrysanthemum indicum based on transcriptome analysis
Source: BMC Genomics. 2018 Apr 25;19:291. doi: 10.1186/s12864-018-4702-1 (PMC5918905; doi:10.1186/s12864-018-4702-1)
Supplement: Supplementary file 1 — Some information extracted from the doctoral thesis about phenotypic difference betwwen two C. indicum germplasms. (DOCX 2701 kb) [file 12864_2018_4702_MOESM1_ESM.docx]

Table Morphological characteristics description of 2 representative *C. indicum* germplasm.

| Germplasm | Collection point | Morphological characteristics |
| --- | --- | --- |
| DIWNT  (diploid) | Luotian, Hubei Province | Incomplete leaves, herbaceous, broad elliptical, with acuminate apex and obcordate base, parted, doubly serrate, 46mm long and 36mm in width; erect stem, green, glabrous, 8.73±0.42 mm in diameter；100.5±5.2 cm in plant height, 91.8±4.4 cm in crown width；yellow head, flowering at the end of October, 12.10±1.12 mm in flower diameter. |
| DIWT  (tetraploid) | Shucheng, Anhui Province | Complete leaves, herbaceous, broad elliptical, with acuminate apex and obcordate base, parted, serrate, 55mm long and 33mm in width; erect stem, fuchsia, covered with hairs, 12.91±0.45 mm in diameter；82.5±4.8 cm in dwarf plant height, 58.2±4.5 cm in crown width；yellow head, flowering in the early November, 15.14±1.39 mm in flower diameter. |

Figure Leaf and stem of 2 representative *C. indicum* germplasm.


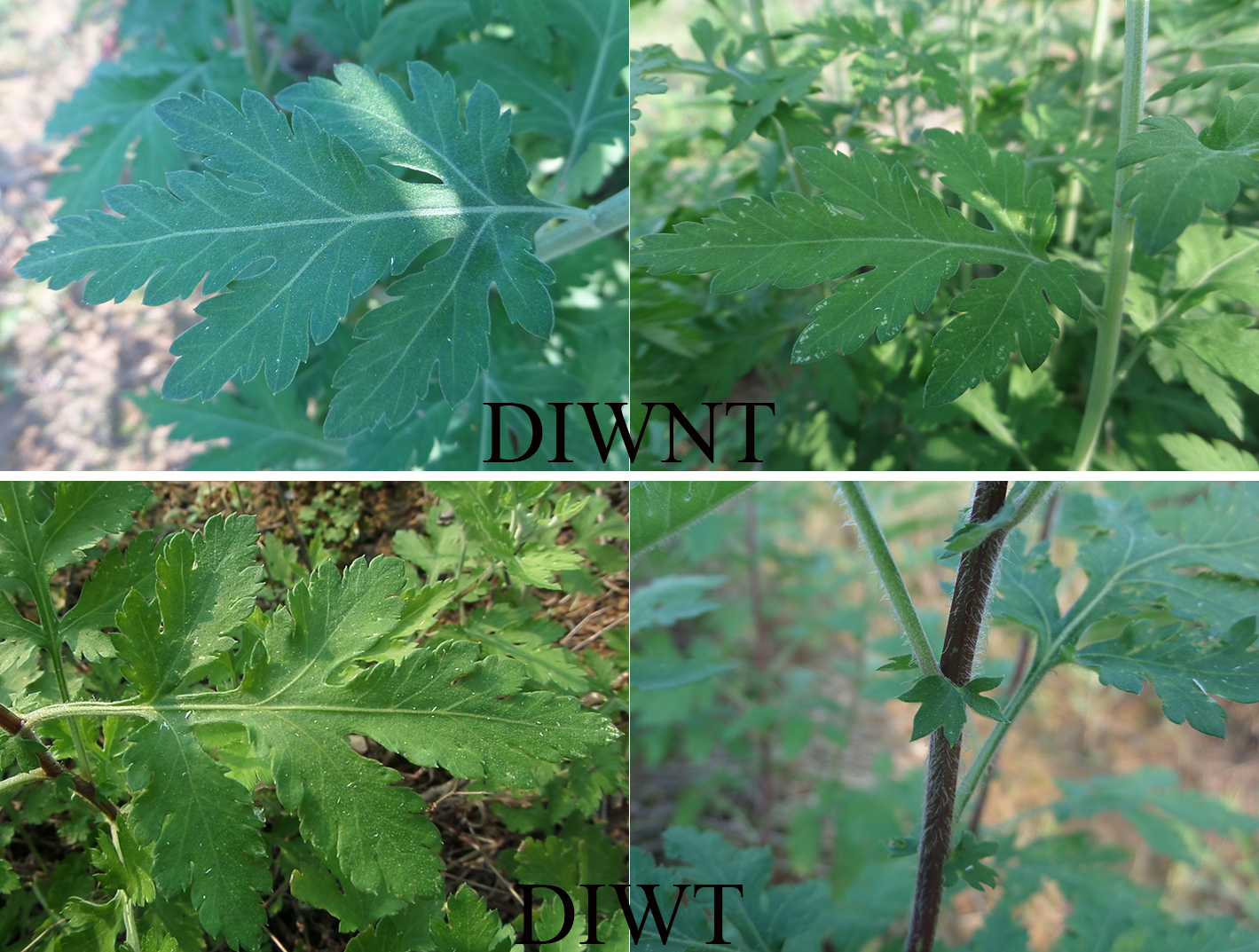


The above information is extracted from the doctoral thesis, “Study on germplasm resources of *Chrysanthemum indicum* L. and quality evaluation of Chrysanthemi Indici Flos. Zhengzhou Han. Guangzhou University of Chinese Medicine, Guangzhou, China. 2017”.
