# Supplementary material for: SSR marker development and intraspecific genetic divergence exploration of Chrysanthemum indicum based on transcriptome analysis
Source: BMC Genomics. 2018 Apr 25;19:291. doi: 10.1186/s12864-018-4702-1 (PMC5918905; doi:10.1186/s12864-018-4702-1)
Supplement: Supplementary file 2 — Table Information for 86 C. indicum samples. (DOC 121 kb) [file 12864_2018_4702_MOESM2_ESM.doc]

| Table Information for 86 *Chrysanthemum indicum* samples | | | | |
| --- | --- | --- | --- | --- |
| Number | Experimental label | Sample source | | Longitude and latitude |
| 1 | FA-1 | Dawu | Hubei Province | E114.12, N31.56 |
| 2 | D01-1 | Ezhou |  | E114.89, N30.40 |
| 3 | F01-1 | Ezhou |  | E114.89, N30.39 |
| 4 | F07-1 | Hong'an |  | E114.61, N31.28 |
| 5 | E02-1 | Huanggang |  | E114.87, N30.43 |
| 6 | F02-1 | Huanggang |  | E114.87, N30.45 |
| 7 | D29-1 | Luotian |  | E115.39, N30.78 |
| 8 | F24-1 | Luotian |  | E115.39, N30.78 |
| 9 | D05-1 | Macheng |  | E115.00, N31.17 |
| 10 | D06-1 | Macheng |  | E115.10, N31.47 |
| 11 | F05-1 | Macheng |  | E114.77, N31.03 |
| 12 | F06-1 | Macheng |  | E114.84, N31.01 |
| 13 | D31-1 | Qichun |  | E115.43, N30.22 |
| 14 | F25-1 | Qichun |  | E115.43, N30.22 |
| 15 | D03-1 | Tuanfeng |  | E114.87, N30.64 |
| 16 | F03-1 | Tuanfeng |  | E114.87, N30.64 |
| 17 | F26-1 | Wuxue |  | E115.56, N29.84 |
| 18 | E04-1 | Xinzhou |  | E114.80, N30.84 |
| 19 | F04-1 | Xinzhou |  | E114.80, N30.84 |
| 20 | FC-1 | Xishui |  | E114.87, N30.45 |
| 21 | D30-1 | Xishui |  | E115.26, N30.45 |
| 22 | E28-1 | Yingshan |  | E115.68, N30.73 |
| 23 | F23-1 | Yingshan |  | E115.68, N30.73 |
| 24 | D12-1 | Guangshan | Henan Province | E111.66, N34.38 |
| 25 | E12-1 | Guangshan |  | E111.66, N34.38 |
| 26 | F10-1 | Guangshan |  | E114.71, N32.14 |
| 27 | D15-1 | Gushi |  | E115.65, N32.16 |
| 28 | F13-1 | Gushi |  | E112.26, N32.45 |
| 29 | E14-1 | Huaibin |  | E115.41, N32.47 |
| 30 | E13-1 | Huangchuan |  | E115.05, N32.13 |
| 31 | F11-1 | Huangchuan |  | E115.05, N32.13 |
| 32 | FB-1 | Luoshan |  | E114.51, N32.20 |
| 33 | D10-1 | Luoshan |  | E114.41, N32.16 |
| 34 | D09-1 | Luoshan |  | E114.51, N32.20 |
| 35 | D16-1 | Shanchen |  | E115.40, N31.79 |
| 36 | F12-1 | Shangcheng |  | E115.40, N31.79 |
| 37 | E07-1 | Xinxian |  | E114.87, N31.64 |
| 38 | D08-1 | Xinxian |  | E114.91, N31.71 |
| 39 | D11-1 | Xixian |  | E114.69, N32.33 |
| 40 | E24-1 | Huaining | Anhui Province | E116.82, N30.73 |
| 41 | F18-1 | Huaining |  | E116.94, N30.72 |
| 42 | F19-1 | Huaining |  | E118.40, N32.46 |
| 43 | D17-1 | Huoqiu |  | E116.27, N32.35 |
| 44 | D20-1 | Huoshan |  | E116.33, N31.39 |
| 45 | F15-1 | Huoshan |  | E116.33, N31.39 |
| 46 | F14-1 | Jinzhai |  | E115.93, N31.72 |
| 47 | E25-1 | Qianshan |  | E116.58, N30.63 |
| 48 | F20-1 | Qianshan |  | E116.57, N30.63 |
| 49 | D18-1 | Shouxian |  | E116.78, N32.57 |
| 50 | D21-1 | Shucheng |  | E116.94, N31.46 |
| 51 | F16-1 | Shucheng |  | E116.94, N31.46 |
| 52 | F21-1 | Taihu |  | E116.30, N30.45 |
| 53 | D22-1 | Tongcheng |  | E116.97, N31.03 |
| 54 | E23-1 | Tongcheng |  | E116.97, N31.03 |
| 55 | F17-1 | Tongcheng |  | E116.97, N31.03 |
| 56 | D19-1 | Yu'an |  | E116.47, N31.73 |
| 57 | D26-1 | Yuexi |  | E116.35, N30.84 |
| 58 | E27-1 | Yuexi |  | E116.01, N30.81 |
| 59 | F22-1 | Yuexi |  | E116.35, N30.79 |
| 60 | C02-1 | Dongyuan | Guangdong Province | E114.74, N23.78 |
| 61 | B01-1 | Pingyuan |  | E115.89, N24.80 |
| 62 | B02-1 | Pingyuan |  | E115.89, N24.80 |
| 63 | B03-1 | Pingyuan |  | E115.89, N24.80 |
| 64 | B04-1 | Pingyuan |  | E115.93, N24.84 |
| 65 | B05-1 | Pingyuan |  | E115.93, N24.84 |
| 66 | B06-1 | Pingyuan |  | E115.93, N24.84 |
| 67 | B07-1 | Pingyuan |  | E115.93, N24.84 |
| 68 | B08-1 | Pingyuan |  | E115.86, N24.79 |
| 69 | B09-1 | Pingyuan |  | E115.86, N24.79 |
| 70 | B10-1 | Pingyuan |  | E115.86, N24.79 |
| 71 | B11-1 | Pingyuan |  | E115.90, N24.81 |
| 72 | B12-1 | Pingyuan |  | E115.90, N24.81 |
| 73 | B13-1 | Pingyuan |  | E115.90, N24.81 |
| 74 | C01-1 | Pingyuan |  | E115.90, N24.81 |
| 75 | A01-1 | Mashan | Guangxi Province | E108.15, N23.70 |
| 76 | A02-1 | Mashan |  | E108.15, N23.70 |
| 77 | A03-1 | Mashan |  | E108.15, N23.70 |
| 78 | A04-1 | Mashan |  | E108.17, N23.70 |
| 79 | A05-1 | Mashan |  | E108.17, N23.70 |
| 80 | A06-1 | Mashan |  | E108.17, N23.70 |
| 81 | A07-1 | Mashan |  | E108.17, N23.70 |
| 82 | A08-1 | Mashan |  | E108.18, N23.65 |
| 83 | A09-1 | Mashan |  | E108.18, N23.65 |
| 84 | A10-1 | Mashan |  | E108.20, N23.61 |
| 85 | A11-1 | Mashan |  | E108.20, N23.61 |
| 86 | A12-1 | Mashan |  | E108.20, N23.61 |
