# Supplementary figures and images for: SSR marker development and intraspecific genetic divergence exploration of Chrysanthemum indicum based on transcriptome analysis
Source: BMC Genomics. 2018 Apr 25;19:291. doi: 10.1186/s12864-018-4702-1 (PMC5918905; doi:10.1186/s12864-018-4702-1)

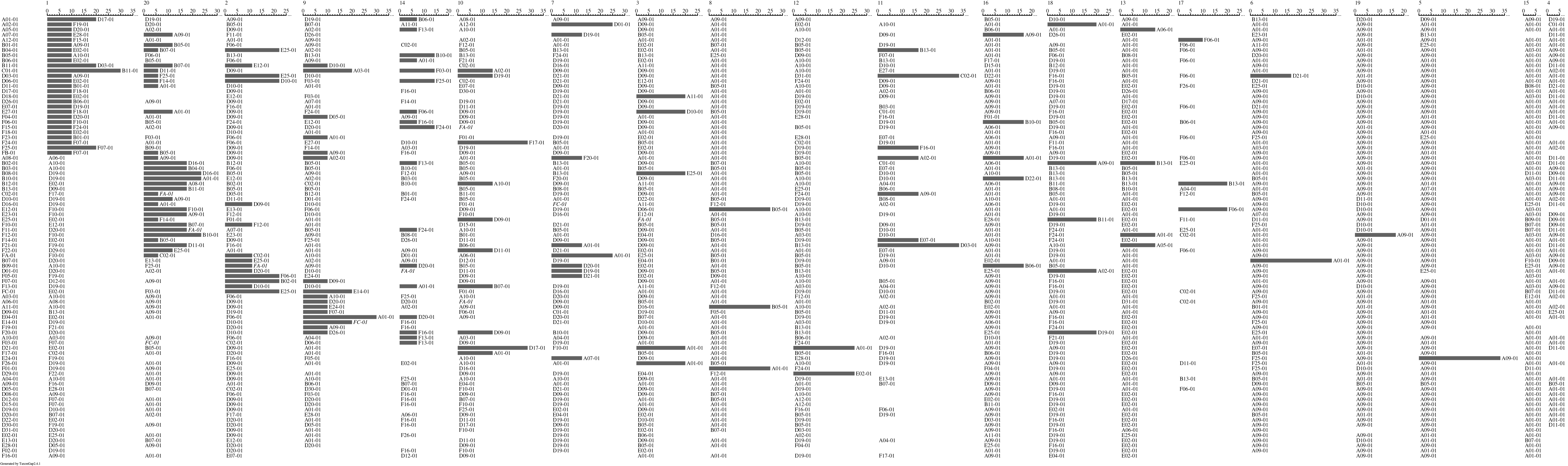

Supplement: Supplementary file 4 — Figure Schematic of identification capacity analysis of 20 SSR primers in all 86 C. indicum germplasm samples. (TIF 1428 kb) [file 12864_2018_4702_MOESM4_ESM.tif]
